# A Noisy Self Goes Online: Intraindividual Personality Variability and Self-Coherence in Digital Engagement

**DOI:** 10.3390/ejihpe16070098

**Published:** 2026-07-09

**Authors:** Giuseppe Forte, Elena Cilloco, Micaela Ambalagi, Ilaria Corbo, Renata Tambelli, Maria Casagrande, Francesca Favieri

**Affiliations:** 1 Department of Dynamic and Clinical Psychology and Health Studies, “Sapienza” University of Rome, Via Degli Apuli 1, 00185 Rome, Italy; elenacilloco7@gmail.com (E.C.); ambalagi.2135961@studenti.uniroma1.it (M.A.); ilaria.corbo@uniroma1.it (I.C.); renata.tambelli@uniroma1.it (R.T.); maria.casagrande@uniroma1.it (M.C.); 2 Department of Psychology, University “G.D’Annunzio” of Chieti and Pescara, Via dei Vestini 30, 66100 Chieti, Italy

**Keywords:** personality traits, coherence in personality, behavioral addictions, maladaptive behaviors

## Abstract

Personality research has traditionally focused on stable trait differences, but increasing attention has been devoted to the internal organization of personality traits within individuals. The present study examined whether intraindividual variability in the Big Five traits, operationalized as the within-person standard deviation across trait scores (iSD_BFI), is associated with problematic digital engagement in young adults. A sample of 316 participants completed the Big Five Inventory and selected subscales of the Behavioral Addiction Questionnaire assessing smartphone use, internet use and video gaming. Pearson correlations, hierarchical regression and independent samples *t*-tests were conducted to evaluate associations between personality trait-profile dispersion and digital behaviors. Results showed that higher iSD_BFI was weakly but significantly associated with greater problematic smartphone use (r = 0.115, *p* = 0.044) and internet use (r = 0.141, *p* = 0.012), whereas no significant association emerged with video gaming (r = 0.029, *p* = 0.764). Hierarchical regression analyses further indicated that iSD_BFI explained a small but statistically significant additional proportion of variance in smartphone use (ΔR^2^ = 0.014, *p* = 0.023) and internet use (ΔR^2^ = 0.019, *p* = 0.008) beyond age, sex, and the five Big Five traits, but not in video gaming. Participants in the problematic smartphone use group exhibited significantly higher iSD_BFI than those in the moderate risk group. Rather than representing a direct measure of self-coherence or identity fragmentation, iSD_BFI was considered an exploratory structural indicator of personality profile dispersion. Accordingly, a more heterogeneous personality profile may be associated with a greater approach to digitally mediated environments, possibly because these environments provide predictability, feedback, and reinforcement. Overall, the study highlights the importance of exploring intraindividual personality configuration as a complementary dimension to traditional trait-based approaches.

## 1. Introduction

Personality has traditionally been conceptualized in terms of stable individual differences captured by broad trait dimensions, such as those defined within the operationalization of the Big Five framework (Costa & McCrae, 1992). This approach helps in describing how individuals differ along core dispositional axes, despite the apparent difficulty in identifying all the features that characterize human beings’ dispositions toward the environment (Epstein, 1994). Also, a critical aspect of personality organization remains comparatively underexplored: the internal configuration of traits within the individual and the extent to which these traits form a coherent versus heterogeneous profile.

Previous studies focused on trait differences with growing evidence highlighting the limits in understanding individuals without considering intraindividual structure (Cervone, 2022). According to the Big Five Model, it appears clear that individuals do not simply possess levels of extraversion, agreeableness, conscientiousness, neuroticism, and openness; rather, these traits coexist within a dynamic configuration that may be differently integrated, affecting emotions, cognition, and behavioral outcomes (McAdams, 1992). Accordingly, two individuals with similar average expressions of the Big Five domains may differ substantially in how they interact with each other, suggesting differences in the structural organization of personality profiles (Cervone, 2005; Fajkowska, 2023). From this perspective, the variability across trait dimensions within the individual can be conceptualized not as a direct measure of self-coherence, but as a structural indicator of the dispersion of trait expression within the personality profile that may be theoretically related to broader aspects of self-organization, without directly measuring the clarity, consistency, or temporal stability of self-representations (Campbell et al., 1996). In the present study, structural personality coherence refers to the relative alignment versus heterogeneity among broad dispositional domains and is considered within theoretical traditions that emphasize the importance of coherence in personality organization. These perspectives range from classic frames on identity integration to contemporary models of self-regulation involving and integrating affective and cognitive processes, despite the functional implications of such structural differences remaining insufficiently understood (Syed & McLean, 2016; Inzlicht et al., 2021, Cervone, 2022; Fajkowska, 2023).

One key question concerns whether a more heterogeneous personality profile, operationalized as greater intraindividual variability across traits, may be associated with differences in how individuals engage with their environment, particularly in domains involving behavioral regulation and reward sensitivity. Recent theoretical developments provide a useful lens for addressing this issue. Within predictive processing accounts of the self, internal models are conceived as hierarchically organized systems that generate predictions about both internal states and external inputs (Woźniak, 2024). A coherent self may correspond to a more stable and precise internal model, whereas a less coherent configuration may reflect increased uncertainty or “noise” in self-representations (Mokady & Reggev, 2022; Tolchinsky et al., 2025) possibly affecting behavioral outcomes.

Interestingly, behaviors involving digitally mediated environments such as smartphone and internet use, which provide continuous accessibility, rapid feedback, immediate reinforcement, and highly structured interaction patterns, may represent a relevant ground on which this hypothesis would be tested. Compared to other potentially problematic behaviors, internet- and smartphone-related activities are deeply embedded in everyday self-regulatory processes and represent pervasive external systems for mood regulation, predictability, and behavioral organization, particularly during young adulthood. Digital environments are characterized by high accessibility, rapid feedback, and structured contingencies, making them well-suited to provide externally regulated input. As such, they may be particularly appealing to individuals showing greater heterogeneity in their dispositional profile, potentially serving a compensatory regulatory function by temporarily reducing uncertainty, enhancing predictability, or providing reward and behavioral organization (Dong & Potenza, 2014; Meng et al., 2015). This interpretation is consistent with the Interaction of Person–Affect–Cognition–Execution (I-PACE) model (Brand et al., 2016, 2019), which conceptualizes problematic internet-related behaviors as the outcome of interactions between predisposing personal characteristics, affective and cognitive responses, and executive control processes. In this sense, repeated reliance on digitally mediated environments for self-regulation may increase the risk of excessive or problematic engagement (Laier et al., 2018).

However, despite these theoretical advances, empirical work linking intraindividual personality structure to behavioral engagement remains scarce. Research has focused mainly on single-trait dimensions, but personality traits do not operate in isolation within the individual but as organized constellations whose combined expression may be more informative (Fleeson, 2001). For example, person-oriented approaches have emphasized that individuals with different configurations of the Big Five traits may display distinct patterns of attitudes and behavior even when mean trait levels appear similar (Bergman & Magnusson, 1997; Asendorpf & Van Aken, 2003; Mõttus et al., 2017; Kerber et al., 2021). Cluster-based studies have shown that profiles characterized by more adaptive combinations of traits differ meaningfully in behavioral tendencies compared with profiles marked by less functional configurations (Poškus & Žukauskienė, 2017). Likewise, research on higher-order Big Five metatraits suggests that shared variance across domains captures broader dispositions toward behavioral engagement (Plasticity) and self-regulatory restraint (stability), indicating that personality structure above the single-trait level may better explain real-world behavior (Hirsh et al., 2009). These findings collectively suggest that examining the internal organization of trait expression may provide a more ecologically valid account of behavioral involvement than considering isolated trait scores alone.

The present study adopts a cautious operational approach to the notion of personality coherence, using the intraindividual standard deviation across Big Five trait scores (iSD_BFI) as an exploratory structural index of personality profile heterogeneity. It is suggested that this index may capture the extent to which broad trait dimensions converge or diverge within the same individual profile. At the same time, profile-based indices require cautious interpretation, because different profiles may exhibit similar levels of dispersion while differing in their specific configurations, psychological meanings, or degrees of normativeness. Because of its exploratory nature, iSD_BFI should not be considered a direct measure of self-coherence, self-concept clarity, or identity integration but rather a reflection of the statistical dispersion of dispositional trait scores. Therefore, higher iSD_BFI values may indicate lower structural alignment among broad personality domains, but they may also reflect differentiation, specialization, or multidimensionality rather than fragmentation per se. Moreover, similar iSD_BFI values may arise from different Big Five configurations and may therefore have different psychological meanings. Accordingly, in the present study, iSD_BFI is interpreted as a quantitative proxy of intraindividual trait-profile heterogeneity, potentially related to structural personality coherence, but not equivalent to self-coherence itself (Cronbach & Gleser, 1953; Furr, 2008; Campbell et al., 1996; Carlier et al., 2024).

Accordingly, the present study aimed to examine whether intraindividual variability in the Big Five traits, operationalized as the within-person standard deviation across trait scores (iSD_BFI), is associated with problematic engagement in digitally mediated behaviors among young adults. It was hypothesized that greater intraindividual variability across the Big Five traits, reflecting a more heterogeneous personality profile and a potentially lower degree of structural trait alignment, would be positively associated with higher levels of problematic digital engagement. Consistent with the exploratory nature of this operationalization, no direct inference was made from iSD_BFI to self-concept clarity, identity fragmentation, or subjective self-coherence. Moreover, we also expected an association between maladaptive trait-level patterns, particularly higher neuroticism and lower conscientiousness and problematic smartphone and internet use.

## 2. Materials and Methods

### 2.1. Participants and Procedure

A sample of 316 young adults (mean age of 22.62 years; SD = 1.79; range = 19–29), of which 224 (70.9%) were women and 87 (27.5%) were men, participated in the study. Participants were recruited through an online survey platform for a cross-sectional study examining the relationship between personality structure and behavioral engagement across multiple domains. Exclusion criteria include diagnoses of psychiatric, neurological or neurodevelopmental disorders. All participants provided informed consent prior to participation, and the study was conducted in accordance with the Declaration of Helsinki and institutional ethical standards. Only participants with complete data on all variables of interest were included in the analyses.

### 2.2. Measures

Personality Traits

Personality traits were assessed using the Big Five Inventory (BFI; John et al., 1991; Italian translation: Fossati et al., 2011), a widely validated self-report instrument measuring five higher-order personality dimensions: extraversion, agreeableness, conscientiousness, neuroticism, and openness to experience. Trait scores were computed following standard scoring procedures, yielding continuous indices for each domain. The Italian validation of BFI (Fossati et al., 2011) reported adequate internal consistency reliabilities (mean α values between 0.77 and 0.81). In our sample good psychometric properties were reported (Cronbach’s α = 0.86; McDonald’s ω = 0.88). For this study, the structural coherence of personality at the individual level was computed as an index of intraindividual variability across the five BFI trait scores. Specifically, for each participant, the standard deviation of the five trait scores was calculated:

$$iSD\text{\_}BFI=\sqrt{\frac{\sum (X_{i}-X{)}^{2}}{N}}$$

where X_i_ represents each trait score, X is the individual’s mean trait level (profile elevation), and N = 5. This metric captures the dispersion of personality traits within an individual profile, independent of overall trait elevation. Higher iSD_BFI values indicate greater heterogeneity across traits, reflecting a less integrated personality configuration, whereas lower values indicate greater internal consistency. In line with prior work on personality profile structure (Fajkowska, 2023), this index can be interpreted as a structural proxy for self-coherence at the trait level, capturing the degree to which personality characteristics converge or diverge within the individual.

Behavioral Domains

The Behavioral Addiction Questionnaire (BAQ; Mastropietro et al., 2022) is a self-report instrument designed to provide a dimensional and quantitative assessment of nine potentially addictive behaviors. It allows each behavior to be screened along a continuum, capturing both adaptive and maladaptive characteristics, and can also yield an overall index of behavioral addiction severity. In the present study, we focused specifically on digital-related behaviors, administering the subscales assessing internet use (α = 0.78), smartphone use (α = 0.77), and video gaming (α = 0.76), in line with the study’s focus on digital-mediated engagement. In our sample good psychometric properties were reported for each scale (internet use: Cronbach’s α = 0.79; McDonald’s ω = 0.80; smartphone use: Cronbach’s α = 0.72; McDonald’s ω = 0.73; video gaming: Cronbach’s α = 0.75; McDonald’s ω = 0.76). Each BAQ subscale consists of 6 items rated on a 6-point Likert scale ranging from 0 (absolutely false) to 5 (absolutely true), yielding subscale scores ranging from 0 to 30, with higher scores indicating more problematic engagement in the target behavior.

### 2.3. Data Preparation and Data Analyses

Descriptive statistics were calculated for personality traits, intraindividual trait-profile dispersion, and digital behavioral measures. Exploratory Pearson’s correlation coefficients were computed to examine the associations among the five Big Five personality traits, intraindividual trait-profile dispersion (iSD_BFI), and smartphone, internet, and video-gaming-related behavioral scores. The iSD_BFI index was interpreted as an exploratory quantitative indicator of the degree of dispersion among an individual’s Big Five trait scores.

To examine whether iSD_BFI provided incremental predictive information beyond demographic characteristics and traditional personality traits, hierarchical multiple linear regression analyses were conducted separately for smartphone use, internet use, and video gaming. Age and sex were entered as demographic covariates in Step 1 (Model 1). Extraversion, agreeableness, conscientiousness, neuroticism, and openness were entered simultaneously in Step 2 (Model 2). Finally, iSD_BFI was entered in Step 3 (Model 3). The incremental contribution of each block was evaluated by examining the change in explained variance (ΔR^2^). The regression coefficients of iSD_BFI in the final models were examined to determine whether trait-profile dispersion remained associated with each behavioral outcome after accounting for age, sex, and the five Big Five traits.

To investigate differences in trait-profile dispersion according to the severity of problematic digital behavior, independent samples *t*-tests were conducted separately for smartphone and internet use. The behavioral risk group, classified according to the BAQ cut-off scores as moderate risk or problematic behavior, was entered as the independent variable, whereas iSD_BFI was entered as the dependent variable. Cohen’s d was calculated as an index of standardized group differences. All statistical tests were two-tailed, and statistical significance was set at *p* < 0.05.

Given the exploratory nature of iSD_BFI statistical significance was interpreted together with effect-size magnitude.

### 2.4. Ethics and AI Adoption

The University Ethics Committee (CERT of “Sapienza” University of Rome) has approved the project (approval date: 5 May 2025; ID: CERT_196c5274271).

Generative artificial intelligence (GenAI) has been used to support text editing (formatting, English editing, and reference organization).

## 3. Results

### Association Between Personality and Behavioral Addiction

Descriptive statistics for the main study variables are reported in Table 1.

Pearson’s correlation analyses were conducted to examine the associations between personality traits, personality configuration (iSD_BFI), and digital behavioral measures (see Table 1). At the level of basic personality traits, results showed a coherent pattern consistent with the literature. Conscientiousness was positively associated with both extraversion (r = 0.224, *p* < 0.001) and agreeableness (r = 0.329, *p* < 0.001) and negatively associated with neuroticism (r = −0.158, *p* = 0.005). Similarly, openness was positively related to all other traits, particularly extraversion (r = 0.367, *p* < 0.001). The intraindividual trait-profile dispersion index (iSD_BFI) showed a positive association between conscientiousness (r = 0.141, *p* = 0.012), agreeableness (r = 0.150, *p* = 0.007) and openness (r = 0.249, *p* < 0.001).

With respect to digital behaviors, problematic smartphone use was negatively associated with extraversion (r = −0.155, *p* = 0.007), agreeableness (r = −0.148, *p* = 0.009), and conscientiousness (r = −0.226, *p* < 0.001) and positively associated with neuroticism (r = 0.377, *p* < 0.001). A similar pattern emerged for internet use, which was negatively related to agreeableness (r = −0.119, *p* = 0.034) and conscientiousness (r = −0.179, *p* = 0.001) and positively associated with neuroticism (r = 0.273, *p* < 0.001). Video gaming was not associated with BFI scales (all r < 0.11, all *p* > 0.12), except for conscientiousness (r = −0.287, *p* = 0.002) which is negatively correlated to the behavior. Importantly, personality configuration coherence (iSD_BFI) was significantly and positively associated with both smartphone use (r = 0.115, *p* = 0.044) and internet use (r = 0.141, *p* = 0.012), but not with video gaming (r = 0.029; *p* = 0.764). Smartphone use, internet use and video gaming were strongly correlated (smartphone–internet: r = 0.750, *p* < 0.001; smartphone–gaming: r = 0.450; *p* < 0.001; internet–gaming: r = 0.378; *p* < 0.001), suggesting a substantial overlap between these dimensions of digital behavior.

Hierarchical linear regression analyses were conducted to examine whether iSD_BFI provided incremental predictive value beyond demographic covariates (Model 1: age, sex) and the traditional Big Five traits (Model 2). For smartphone use, the model including age and sex explained 3.0% of the variance (R^2^ = 0.030). Adding the Big Five traits in Step 2 significantly improved model fit, ΔR^2^ = 0.183, *p* < 0.001 (R^2^ = 0.213; R^2^_adj_ = 0.189). The addition of iSD_BFI in Step 3 explained a small but statistically significant additional proportion of variance, ΔR^2^ = 0.014, *p* = 0.023 (R^2^ = 0.226; R^2^_adj_ = 0.200). In the final model, iSD_BFI was positively associated with smartphone use, B = 0.648, SE = 0.283, t = 2.29, *p* = 0.023.

For internet use, Model 1 explained 4.8% of the variance (R^2^ = 0.048). Adding the Big Five traits significantly improved model fit, ΔR^2^ = 0.113, *p* < 0.001 (R^2^ = 0.161; R^2^_adj_ = 0.137). Model 3 explained a small but statistically significant additional proportion of variance, ΔR^2^ = 0.019, *p* = 0.008 (R^2^ = 0.180; R^2^_adj_ = 0.153) with iSD_BFI was positively associated with internet use, B = 0.870, SE = 0.328, t = 2.66, *p* = 0.008.

For video gaming, the model including age and sex explained 2.4% of the variance (R^2^ = 0.024). Adding the Big Five traits in Step 2 significantly improved model fit, ΔR^2^ = 0.136, *p* = 0.009 (R^2^ = 0.160; R^2^_adj_ = 0.093). However, the addition of iSD_BFI in Step 3 did not explain additional variance, ΔR^2^ < 0.001, *p* = 0.869. Overall, these analyses indicate that iSD_BFI explained a small but statistically significant amount of additional variance in smartphone and internet use beyond age, sex, and Big Five traits, whereas no incremental contribution emerged for video gaming.

**Figure 1:**
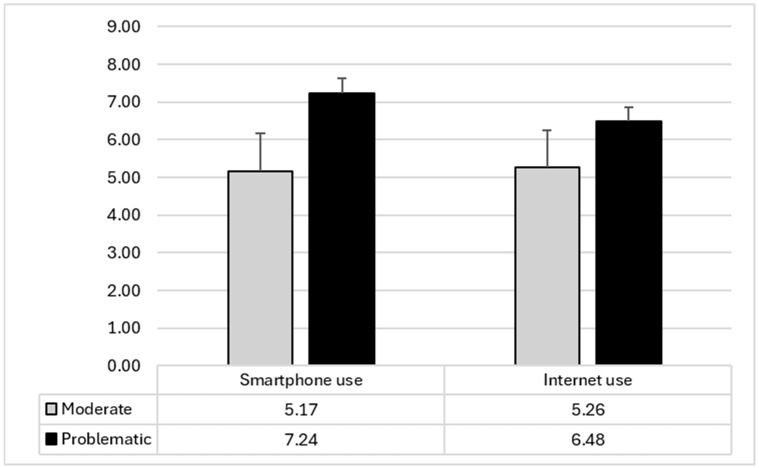
*T*-tests of behavior type (moderate risk vs. problematic) on iSD_BFI score.

Considering BAQ cut-off scores indicating different degrees of behavioral risk, *t*-test analyses were conducted with group as the independent variable (moderate risk behavior; problematic behavior) and iSD_BFI as the dependent variable, with results showing a significant difference between groups in smartphone use (t = −2.32; *p* = 0.03; Cohen’s d = −0.97; see Figure 1), highlighting higher heterogeneity in personality configuration in the problematic behavior group. Conversely no significant difference was found between the problematic behavior group compared to the moderate risk group in internet use (t = −1.10; *p* = 0.28; Cohen’s d = −0.52).

## 4. Discussion

One of the main contributions of this study was to propose a strategy for operationalizing and quantitatively evaluating intraindividual heterogeneity in the Big Five personality profiles, in which not only mean trait levels but also the internal configuration of traits is considered informative. By focusing on intraindividual variability, the analysis captures an aspect of personality organization that is not accessible through traditional variable-centered approaches. In this framework, iSD_BFI was interpreted as an exploratory structural index of trait-profile dispersion. Findings suggest that problematic digital engagement is associated not only with specific personality traits but also, to a limited extent, with the organization of personality, as captured by an index reflecting the degree of heterogeneity across personality traits. In line with the previous literature, higher levels of neuroticism and lower levels of conscientiousness were associated with increased problematic smartphone and internet use, supporting the role of trait-level vulnerabilities in digital dysregulation (Marciano et al., 2020, 2022; Hidalgo-Fuentes & Fernández-Castilla, 2024; Eichenberg et al., 2021; Müller et al., 2021; Forte et al., 2023). Greater intraindividual trait-profile dispersion was also positively associated with smartphone and internet use, although the magnitude of these correlations was small. No significant association emerged between iSD_BFI and video-gaming scores, suggesting that the relevance of trait-profile dispersion may not generalize uniformly across different forms of digital behavior. Hierarchical regression analyses, controlled for age and sex, further showed that iSD_BFI explained a small but statistically significant additional proportion of variance beyond the Big Five traits for smartphone and internet use, but not for video gaming. These findings suggest that iSD_BFI may provide modest complementary information beyond demographic characteristics and individual trait levels for understanding problematic smartphone and internet engagement. From this perspective, the result is consistent with a view of personality as a coordinated system, in which the relative alignment among traits may contribute to the stability and predictability of behavior (Mischel & Shoda, 1995). A more aligned personality configuration may facilitate consistent goal-directed action, clearer prioritization, and more efficient self-regulation. In contrast, greater heterogeneity may reflect a configuration in which behavioral tendencies are less aligned, potentially resulting in increased variability and reduced regulatory stability (Campbell et al., 2003), supporting the relevance of personality profile organization in defining the features of behaviors. Nevertheless, given the small power of the observed associations and the modest ΔR^2^ values, these results should be interpreted as preliminary evidence for the complementary relevance of trait-profile organization, rather than as evidence that iSD_BFI is a major determinant of problematic digital engagement. Rather, iSD_BFI should be considered as one complementary indicator among several dispositional, affective, cognitive, and contextual factors that may contribute to digital behavior (Brand et al., 2016, 2019; Billieux et al., 2015; Marengo et al., 2020; Gioia et al., 2021; Yan et al., 2022).

In this sense, iSD_BFI can be interpreted as an exploratory index of intraindividual personality profile organization, complementing traditional trait-based approaches. From a self-regulation perspective, individuals showing greater heterogeneity in their dispositional profile may theoretically be more likely to rely on external structures to support regulation, particularly in contexts characterized by high demands on attention or affective control (Carver & Scheier, 2012). Digital environments, such as smartphones and internet-based platforms, provide continuous access to structured, responsive, and highly reinforcing stimuli (Billieux et al., 2015). These features may make them particularly suitable as external support for regulating attention and emotional states, especially although this mechanism was not directly tested in the present study (Brand et al., 2014). However, given the cross-sectional and self-report nature of the present study, this interpretation should be considered a theoretical hypothesis rather than an established explanatory mechanism. The association between iSD_BFI and digital behaviors can also be interpreted within broader models of predictive and regulatory functioning, in which the self is conceptualized as a system that generates and updates internal models to guide behavior. A more structurally aligned dispositional profile may be theoretically related to more stable patterns of self-regulation, whereas greater heterogeneity among broad trait domains may be associated with increased variability in regulatory tendencies. In this context, digital platforms may serve as environments that reduce uncertainty by offering rapid, contingent responses, thereby supporting short-term regulation. However, this interpretation should be tested in further longitudinal or experimental studies to reduce its speculative feature. Due to these characteristics, engagement in digitally mediated activities, which at the beginning is experienced as inherently pleasurable and rewarding, could transition to problematic usage if the media consumption behavior starts to act as an important or exclusive mechanism to relieve stress, loneliness, depression, or anxiety, providing a means of escape, or as a way to develop a feeling of mastery (Tokunaga, 2015). In fact, individuals who frequently use maladaptive regulatory strategies, such as avoidance and rumination, are more prone to use internet activities to cope with negative affect, potentially leading to the development of internet addiction (Yan et al., 2022). However, further studies should explore this dimension that was not considered in the current work.

The developmental characteristics of the sample are also relevant for interpreting these findings. The participants were predominantly Italian young adults, a phase often associated with ongoing processes of identity consolidation and self-organization (Arnett, 2000). During this period, variability across traits may reflect not only stable individual differences but also transitional configurations, in which different aspects of the self are still being integrated. Moreover, because the sample was likely characterized by relatively homogeneous educational and university-related experiences, the findings should be interpreted within this specific sociocultural and developmental context. In such a context, digital environments may serve as both spaces for exploration and as sources of external structure, potentially interacting with individual differences in trait-profile organization (Peter & Valkenburg, 2006).

The strong association between smartphone and internet use suggests that these behaviors are closely related and may reflect a shared underlying dimension of digital engagement rather than entirely distinct behaviors. This supports a more integrated conceptualization of digital use, in which different platforms contribute to a broader pattern of interaction with technology. At the same time, the weaker associations involving video gaming and the absence of an incremental contribution of iSD_BFI for this domain suggest that different forms of digital behavior should not be considered fully interchangeable. Gaming may involve partially distinct motivational, social, and behavioral processes that are not captured by the same personality profile characteristics associated with smartphone and internet use.

Despite the new perspective suggested by the study, some limitations should be noted. First, the cross-sectional design does not allow for causal conclusions, and it remains unclear whether greater trait-profile heterogeneity contributes to increased digital engagement or whether sustained patterns of digital use may influence the organization of personality over time. For this reason, interpretations involving predictive processing, uncertainty reduction, and external regulatory support should be considered theoretical hypotheses rather than empirically established mechanisms. Moreover, the associations involving iSD_BFI were small. Therefore, the practical significance of iSD_BFI should be interpreted cautiously. Second, all variables were assessed through self-report instruments, which may introduce response biases, social desirability effects, and shared measurement variance. Also, the study did not assess actual time spent using digital technologies. Consequently, it was not possible to determine whether the observed associations were specifically related to problematic engagement or partly reflected the overall frequency or intensity of use. Future studies should integrate multi-method assessments, including behavioral indicators of digital use, informant reports, ecological momentary assessment, or objective smartphone use data. Third, the sample was characterized by a sex imbalance and was restricted to a specific age group and demographic group (university students). Future research should replicate these findings in larger, more balanced, and more heterogeneous samples.

A further limitation concerns the operationalization of personality coherence through iSD_BFI. Although this index provides a quantitative description of intraindividual trait-profile heterogeneity, it should not be interpreted as a direct measure of self-coherence. Similar iSD_BFI values may arise from different Big Five configurations and may therefore reflect different psychological meanings. For example, greater dispersion may indicate specialization or fragmentation depending on the specific trait configuration and broader psychological context. Moreover, profile-based indices may not capture subjective, developmental, or narrative dimensions of self-organization. Therefore, the present findings should be interpreted as preliminary evidence linking trait-profile heterogeneity to problematic digital engagement, rather than direct evidence that reduced self-coherence or identity fragmentation directly underlies such behaviors. Future studies should combine iSD_BFI with validated measures of self-concept clarity, identity coherence, or personality functioning, to better establish its convergent and discriminant validity. Moreover, integrating additional measures of self-organization, such as indices of identity coherence, intraindividual variability, interoceptive processes, or indices of autonomic dynamics (i.e., heart rate variability) (Troisi et al., 2026), may help clarify the mechanisms underlying these associations. Moreover, replication in more balanced and methodologically diverse samples is required before stronger theoretical, predictive, or clinical conclusions can be drawn.

In summary, the present study highlights the relevance of considering personality configuration, in addition to individual traits, in understanding problematic digital behaviors. Although these findings should not be interpreted as indicating that trait-profile heterogeneity is a major determinant or diagnostic marker of problematic digital engagement, a more heterogeneous Big Five personality profile appears to be associated with higher levels of digital engagement. This preliminary evidence, despite the small magnitude of the effect requiring a more cautious interpretation, suggests that the organization of personality traits, as a possible expression of the self, may represent an important dimension in the study of technology-related behaviors.

## Figures and Tables

**Table 1:** Descriptive information of the sample.

Variable	Mean (SD)
Age	22.62 (1.79)
Extraversion	25.51 (5.30)
Agreeableness	32.82 (5.22)
Conscientiousness	32.29 (6.47)
Neuroticism	26.30 (6.11)
Openness	36.21 (6.79)
Total BFI score	21.72 (14.89)
iSD_BFI	5.60 (2.12)
Smartphone use	6.74 (4.39)
Internet use	9.02 (4.99)
Video gaming	8.19 (5.13)

## Data Availability

Raw datasets are available upon reasonable request to the corresponding authors.
